# Surface Reconstruction on Electro-Spun PVA/PVP Nanofibers by Water Evaporation

**DOI:** 10.3390/nano12050797

**Published:** 2022-02-26

**Authors:** Feipeng Wang, Zheng Zhang, Yuyang Yan, Zijia Shen, Qiang Wang, Reimund Gerhard

**Affiliations:** 1 State Key Lab of Power Transmission Equipment & System Security and New Technology, Chongqing University, Chongqing 400044, China; 201911021006@cqu.edu.cn (Z.Z.); 20143232@cqu.edu.cn (Y.Y.); shenza@mail.uc.edu (Z.S.); wangqiang0609@cqu.edu.cn (Q.W.); 2 Institute of Physics and Astronomy, Faculty of Science, University of Potsdam, 14476 Potsdam-Golm, Germany; reimund.gerhard@uni-potsdam.de

**Keywords:** surface reconstruction, intermolecular force, surface-roughened nanofiber

## Abstract

Tailoring the secondary surface morphology of electro-spun nanofibers has been highly desired, as such delicate structures equip nanofibers with distinct functions. Here, we report a simple strategy to directly reconstruct the surface of polyvinyl alcohol/polyvinylpyrrolidone (PVA/PVP) nanofibers by water evaporation. The roughness and diameter of the nanofibers depend on the temperature during vacuum drying. Surface changes of the nanofibers from smooth to rough were observed at 55 °C, with a significant drop in nanofiber diameter. We attribute the formation of the secondary surface morphology to the intermolecular forces in the water vapor, including capillary and the compression forces, on the basis of the results from the Fourier-transform infrared (FTIR) and X-ray photoelectron (XPS) spectroscopy. The strategy is universally effective for various electro-spun polymer nanofibers, thus opening up avenues toward more detailed and sophisticated structure design and implementation for nanofibers.

## 1. Introduction

The applications of nanofibers in air and water filtration [1], for catalyzing substrates [2], in various sensors [3], as biomedical scaffolds [4,5] and drug carriers [6] are rapidly increasing, thanks to their much larger surface area. It is well recognized that distinctive secondary morphologies or structures, as found in, e.g., core-shell nanofibers [7], hollow nanofibers [8], triaxial nanofibers [9], tree-like nanofibers [10], surface-roughened nanofibers, etc., can significantly enhance functionality and efficiency. Surface-roughened or grooved nanofibers with an easy-to-prepare secondary morphology are receiving fast increasing attention, as they offer useful interactions with surrounding objects at a low cost. Consequently, they are being implemented at a fast pace into highly cell-attachable scaffolds [11], optimized ethylene-glycol gas sensors [12], stronger bonded construction materials [13], etc. Several routes, such as chemical corrosion or selective dissolution, are feasible for obtaining rough or grooved nanofiber surfaces. Due to their high biocompatibility, polyvinyl alcohol (PVA) nanofibers are very suitable as a polymer matrix for cell-attachable scaffolds. However, it is challenging to achieve sufficiently high roughness on PVA-nanofiber surfaces in order to anchor cells, gas molecules, or cement-hydration products because of the rather low surface tension of PVA solutions, which usually forces the formation of very smooth nanofiber surfaces during electrospinning. Low surface tension and high water-vapor permeability are found to be responsible for preventing the formation of stable secondary structures through surface reconstruction [14,15]. It has been shown that parameters such as solution concentration, externally applied voltage, flow rate, collecting distance, etc., during electrospinning are not suitable for preparing PVA fibers with the required secondary morphologies [16]. Consequently, there is a strong motivation to develop well-controlled and efficient methods to achieve high levels of surface reconstruction on PVA nanofibers.

In this work, we report secondary-morphology formation on PVA nanofibers, which was achieved via surface reconstruction of electrospun PVA/polyvinylpyrrolidone (PVP) nanofibers by means of water evaporation in a vacuum. The process was enhanced by selective dissolution as the last step.

## 2. Materials and Methods

**Figure 1:**
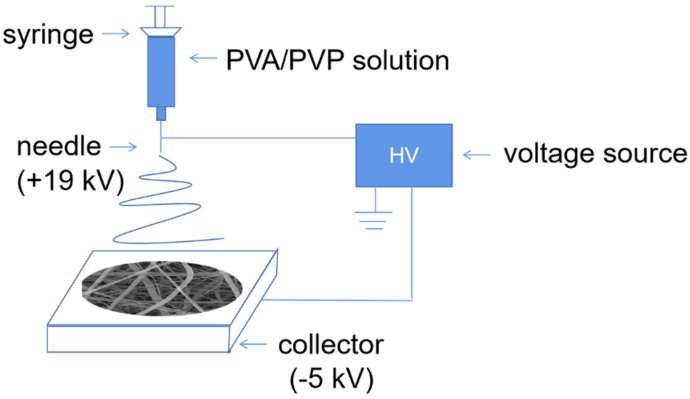
Setup of the experimental electrospinning apparatus.

The PVA powder, specified as #1799 (Mw = 74,885) from Aladdin Co. Ltd., Shanghai, China, was selected because it is soluble in hot water (typically at temperatures above 95 °C), but not dissolvable in water at RT (room temperature, RT~25 °C), and slightly dissolvable in warm water (e.g., at 55 °C). The PVP powder, specified as #K30 (Mw = 44,000~54,000) from Chron Co. Ltd., Chengdu, China, was employed as the second polymer because of its significant solubility in both water and ethanol—regardless of temperature. Both polymer powders have purities better than 99%. First, 3 g PVA and 3 g PVP were mixed and dissolved in 34 g of ultra-pure water with a resistivity higher than 1.8 G Ω·m at 25 °C. Electro-spinning of the PVA/PVP nanofibers was carried out at a nozzle voltage of +19 kV and a collector voltage of −5 Kv (cf. Figure 1) on the electrospinning machine (WL-2, Aibo Zhiye Ion Technology Limited Company, Beijing, China). The flow rate of 0.5 mL/h and the distance of 24 cm between nozzle and collector were controlled by means of a syringe pump and a 3D controller. Nanofibers used immediately after electro-spinning were labeled as V samples and needed further processing due to their softness and sticky surface. Therefore, the V samples were subjected to 4 h vacuum drying either at RT (labeled as VRT) or at 55 (V55), 75 (V75), or 95 °C (V95) at vacuum drying chamber (DZF-6050, CIMO Medical Instruments Co., Shanghai, China). In order to obtain PVA nanofibers, the four types of samples were immediately treated in 500 mL ethanol for 48 h with magnetic stirring at 200 rad/min to remove PVP by means of selective dissolution, and ethanol residues were removed via RT drying at 100 Pa. The samples that had been selectively dissolved were labeled as VRT-SD, V55-SD, V75-SD, and V95-SD. The followed Scanning Electron Microscopy (SEM; Quattro S, Thermo Fisher Scientific, Waltham, MA, USA) and Energy-Dispersive X-ray observations were carried out with a high degree of vacuum (1 × 10^−^^3^ Pa) at 25 °C. The XPS (ESCALAB250Xi, Thermo Fisher Scientific, Waltham, MA, USA) test was carried out at 5 × 10^−^^8^ Pa and 25 °C. The FTIR (Alpha, Bruker, Germany) test was carried out at 25 °C.

## 3. Results

**Figure 2:**
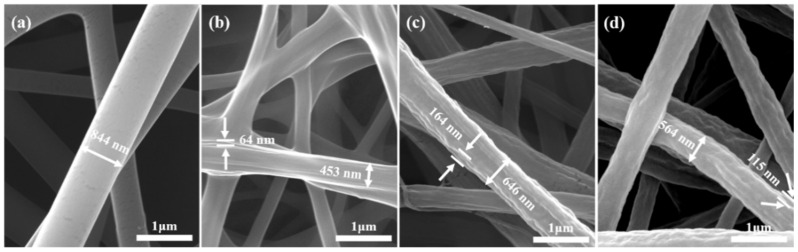
SEM photographs of samples (**a**) V, (**b**) VRT-SD, (**c**) V55-SD, and (**d**) V95-SD, with nanofiber diameters and surface-roughness values as indicated.

The samples in Figure 2 show the nanofiber morphologies, as seen in SEM. Sample V is quite uniform and smooth, and there are no observable pores or stratifications (Figure 2a). Vacuum drying at RT, 55 °C, 75 °C, or 95 °C brought no observable change to the surface morphology, as seen via SEM (figures not shown). However, after selective dissolution of PVP, all nanofiber samples (VRT-SD, V55-SD, V75-SD, and V95-SD) exhibited significantly different surface morphologies. Sample VRT-SD showed a quite noticeable roughness of ~64 nm and a remarkably reduced fiber diameter (from 844 to 453 nm, cf. Figure 2a,b). Sample V55-SD exhibited rough fiber surfaces with obvious grooves and depressions. The roughness was estimated as 164 nm, and the fiber diameter was found to only be reduced from 844 to 646 nm (cf. Figure 2a,c). The nanofiber morphologies of samples V75-SD and V95-SD were similar, so that the SEM image of V75-SD does not have to be shown here. The surface of sample V95-SD showed obvious roughness with some grooves (cf. Figure 2d), but the roughness was lower than that of sample V55-SD and higher than that of sample VRT-SD. In agreement with the roughness variation, the diameters of V75-SD and V95-SD were found to be between those of samples VRT-SD and V55-SD. 

**Figure 3:**
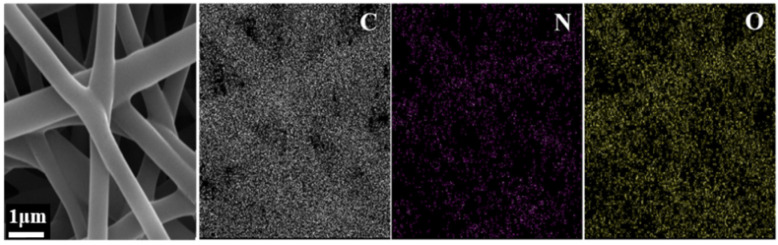

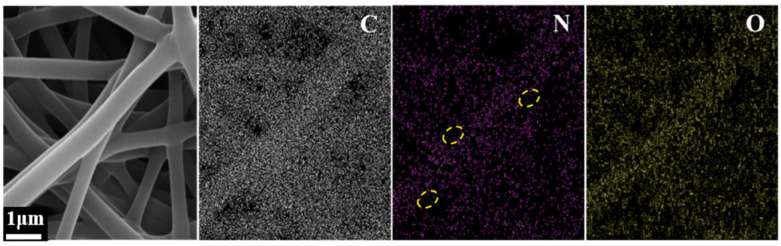
EDX element-distribution maps of samples VRT (**top** panel) and V55 (**bottom** panel).

The two panels in Figure 3 show the element distributions in samples VRT and V55 obtained by means of EDX mapping. As expected, both samples contained Carbon, Oxygen, and Nitrogen. Vacuum drying at RT led to homogeneous distributions of C, O, and N on the nanofiber surfaces of sample VRT (top panel of Figure 3). Vacuum drying at the elevated temperature of 55 °C yielded quite a different element distribution for sample V55. The bottom panel of Figure 3 reveals a homogeneous distribution of C and O, but a heterogeneous distribution of N on the nanofiber surface of sample V55. The black regions enclosed by yellow ellipses near the nanofiber contours indicate a lack of Nitrogen and thus a nonuniform overall concentration of PVP. The concentration variation of the PVP resulted from the morphology variations between samples VRT and V55, as the surface roughness of V55 was achieved through the removal of the PVP in ethanol. 

## 4. Discussion

**Figure 4:**
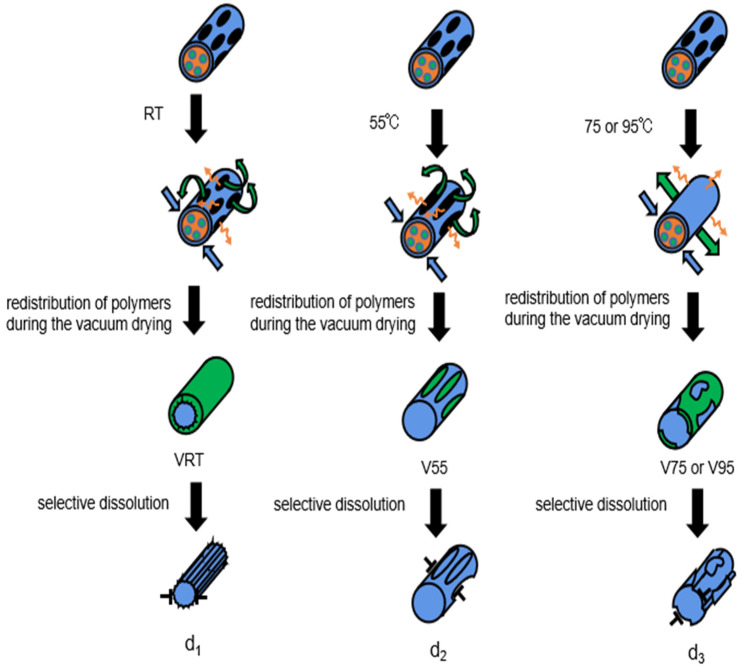
Surface reconstruction process during vacuum drying at various temperatures (Green, blue, orange, and black regions on the fiber represent PVP, PVA, water, and mesh voids, respectively. The green, blue, and orange arrows in the diagram represent the traveling directions of PVP, PVA, and water, respectively).

The observations suggest a hypothesis (schematically shown in Figure 4) for the surface reconstruction during vacuum drying at either RT or elevated temperatures. The surface morphologies can be traced back to the electro-spinning process, in which electrostatic repulsion of surface charges deforms each PVA/PVP droplet into a Taylor cone. The charged jet ejected from the Taylor cone is stretched and split into new finer jets, so that water evaporates quickly and leaves PVA as the polymer to crystallize first, if the very high solubility of PVP in water is taken into account. On the other hand, PVP and the remaining water still exist as a concentrated solution inside the nanofibers, as evidenced from the high softness and stickiness of sample V. Therefore, the inner-liquid and outer-crystallizing process can be reasonably assumed to yield to nanofiber shells that are metastable, mesh-like PVA layers [17]. The subsequent vacuum drying at RT allows the internal water in the fibers to carry PVP molecules uniformly from the inside to the outside through the PVA mesh-like layers. Capillary forces from the PVP/water migration and compression forces from the water evaporation can lead to significant cracking and shrinkage of the PVA mesh-like layers. Thus, after the PVP removal by selective dissolution, a significant reduction of the nanofiber diameter and a slightly wrinkled nanofiber surface can be expected—as seen in the SEM image of sample VRT-SD in Figure 2b. In the case of vacuum drying at the elevated temperature of 55 °C, the mesh-like PVA layer is thermally softened. Therefore, the capillary force from water migration leads to deformation instead of cracking in the PVA mesh. The following water evaporation boosts the formation of this undulating profile on the PVA layer. As a result, after selective dissolution, the nanofiber has no obvious reduction in diameter but exhibits a significant increase in rough surfaces (cf. Figure 2c). 

As mentioned above, further elevated temperatures of 75 or 95 °C during vacuum drying of the V75-SD and V95-SD samples led to smaller morphology changes than in samples VRT-SD and V55-SD. Upon exposure to the two higher temperatures, PVA tends to be quickly dissolved in water together with PVP, which produces some PVA fragments. Hence, the capillary force is strongly reduced, but the compression force from the water evaporation generates an axial motion of PVA fragments, as indicated in Figure 2d, instead of a radial crash or deformation of the PVA mesh-like layer. This is supported by the SEM images in Figure 2d.

**Figure 5:**
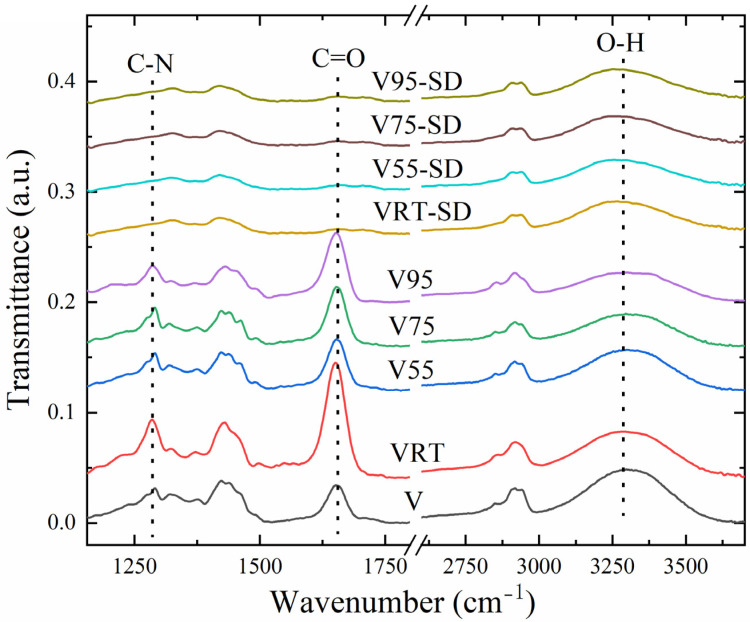
FTIR spectra of the nanofibers in all 9 differently prepared and treated samples.

The water evaporation is evident from the results of Fourier-Transform Infrared (FTIR) and X-ray Photoelectron Spectroscopy (XPS) scans. In Figure 5, the FTIR peaks at 1660 and 1290 cm^−1^ are attributed to C=O and C-N bonds in ketone, respectively, and the broad peak at ~3300 cm^−1^ indicates hydrogen bonds [18,19]. Taking sample V as a reference, the PVP content, quantifiable by the signature peak for C=O bonds at 1660 cm^−1^, shows an increase, followed by a decrease and a second increase, i.e., changes to 197%, 31%, 51%, and 74%, respectively, for VRT, V55, V75, and V95. 

Beside water, internal hydrogen bonds in both polymers also contributed to the FTIR peak at ~3300 cm^−1^. We assume that the sample V95 represents only PVA, since there is no reason for water molecules to remain in the sample. The prominent O–H peak at ~3300 cm^−1^ indicates a considerable water content for sample V. When the vacuum-drying temperature increases from RT to 95 °C, this leads to increasing water losses—as indicated by a strong reduction of the peak starting at ~3100 cm^−^^1^ (cf. the FTIR curves for samples VRT, V55, V75, and V95). 

The selective dissolution of the PVP rendered the FTIR spectra of samples VRT-SD, V55-SD, V75-SD, and V95-SD almost identical. As expected, the peaks for C–N (1660 cm^−1^) and C=O (1290 cm^−1^) are not visible, implying the absence of PVP because of the selective dissolution. We notice, however, that selective dissolution has slightly impaired the broad peak for hydrogen bonding at 3300 cm^−1^, which probably indicates a lower hydroxyl concentration due to the loss of PVP during selective dissolution. 

**Figure 6:**
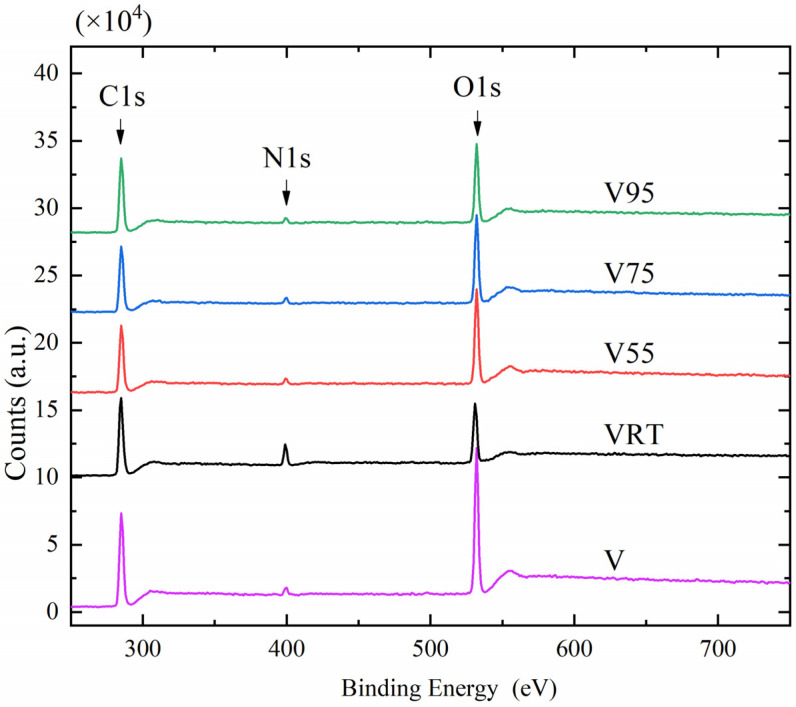
XPS spectra of nanofiber samples (Peaks of C, O, N are marked out).

XPS, a highly sensitive method for surface analysis with a typical probing depth of ~5–10 nm, is highly feasible for investigating the near-surface composition in nanofibers. As indicated in Figure 6, the peaks at 285, 399, and 531 eV represent the C1s, N1s, and O1s orbitals, respectively [20]. The high O content (32.22%) in sample V confirms the existence of a PVA shell for the nanofibers because PVA features the highest intrinsic O content (~36%). Under the assumption that PVA and PVP were both uniformly distributed with a 50/50 (wt./wt.) ratio, the atomic ratio of N is 6.43%. In Figure 6, the atomic ratios of N for samples V, VRT, V55, V75, and V95 are found to be 3.14%, 9.90%, 3.66%, 3.99%, and 4.04% respectively. The N content in V55, V75, and V95 is similar, but exhibited a slight increase with temperature, which means that 55 °C is the critical vacuum-drying temperature to yield a uniform distribution of PVA and PVP on nanofiber surfaces. The slightly increased N content in samples V75 and V95 was due to a corresponding decrease of O, because more bound water was eliminated at 75 °C and 95 °C compared to 55 °C. The results support the hypothesis that vacuum drying at RT leads to PVP-rich nanofiber surfaces, while vacuum drying at 55, 75, and 95 °C yields the opposite result, i.e., a PVA-rich surface. It is obvious that the XPS results agree well with the FTIR observations. The XPS spectra indicate that more PVA was collected on the nanofiber surfaces of these samples (notice the O1s peak at 531 eV increases sharply for sample V55, because each PVA molecule has a hydrogen bond, while PVP does not).

## 5. Conclusions

In conclusion, reconstruction of PVA/PVP nanofiber surfaces can be achieved and controlled by water evaporation during vacuum drying at selected temperatures. The intermolecular forces induced by the water evaporation are recognized as the drivers of the process. It was found that vacuum drying at 55 °C equips the nanofibers with significant surface roughness due to the combined effects of the capillary forces from water migration and the compression forces from water evaporation, a process that has been proven to be a feasible route for obtaining a high roughness on PVA nanofibers and, thus, for enabling specific applications that require nanofibers with nano-structured surfaces. 

## Data Availability

The data that support the findings of this study are available from the corresponding author upon reasonable request.
